# High energy density rechargeable magnesium battery using earth-abundant and non-toxic elements

**DOI:** 10.1038/srep05622

**Published:** 2014-07-11

**Authors:** Yuki Orikasa, Titus Masese, Yukinori Koyama, Takuya Mori, Masashi Hattori, Kentaro Yamamoto, Tetsuya Okado, Zhen-Dong Huang, Taketoshi Minato, Cédric Tassel, Jungeun Kim, Yoji Kobayashi, Takeshi Abe, Hiroshi Kageyama, Yoshiharu Uchimoto

**Affiliations:** 1 Graduate School of Human and Environmental Studies, Kyoto University, Yoshida-nihonmatsu-cho, Sakyo-ku, Kyoto 606-8501, JAPAN; 2 Office of Society-Academia Collaboration for Innovation, Kyoto University, Gokasho, Uji, Kyoto 611-0011, JAPAN; 3 Graduate School of Engineering, Kyoto University, Katsura-cho, Nishikyo-ku, Kyoto 615-8510, JAPAN; 4 The Hakubi Center for Advanced Research, Kyoto University, Yoshida-Ushinomiya-cho, Sakyo-ku, Kyoto 606-8302, JAPAN; 5 Japan Synchrotron Radiation Research Institute, 1-1-1 Kouto, Sayo-cho, Sayo-gun, Hyogo 679-5198, JAPAN; 6 Institute for Integrated Cell-Material Sciences, Kyoto University, Yoshida-Ushinomiya-cho, Sakyo-ku, Kyoto 606-8302, JAPAN

## Abstract

Rechargeable magnesium batteries are poised to be viable candidates for large-scale energy storage devices in smart grid communities and electric vehicles. However, the energy density of previously proposed rechargeable magnesium batteries is low, limited mainly by the cathode materials. Here, we present new design approaches for the cathode in order to realize a high-energy-density rechargeable magnesium battery system. Ion-exchanged MgFeSiO_4_ demonstrates a high reversible capacity exceeding 300 mAh·g^−1^ at a voltage of approximately 2.4 V *vs.* Mg. Further, the electronic and crystal structure of ion-exchanged MgFeSiO_4_ changes during the charging and discharging processes, which demonstrates the (de)insertion of magnesium in the host structure. The combination of ion-exchanged MgFeSiO_4_ with a magnesium bis(trifluoromethylsulfonyl)imide–triglyme electrolyte system proposed in this work provides a low-cost and practical rechargeable magnesium battery with high energy density, free from corrosion and safety problems.

Rechargeable batteries have become quintessential energy conversion devices, that are widely used in portable electronic devices and hybrid electric vehicles. However, their energy density and safety still require improvement, particularly considering their future demand as larger power sources for electric vehicles and smart grid communities^1^. Rechargeable magnesium metal batteries are one potential solution. As an anode, magnesium metal provides two electrons per atom, giving it an attractive volumetric capacity of 3837 mAh·cm^−3^, which is approximately five times higher than that of the conventional graphite anodes in lithium ion batteries (LIBs). In addition to the high capacity, the relatively high negative reduction potential of magnesium metal can provide high energy density. Moreover, the terrestrial abundance and melting point of elemental magnesium by far surpass that of lithium, translating to a cheap and safe battery system. These advantages of magnesium metal anodes have been previously recognized^2^,^3^, and a rechargeable magnesium battery cell was first proposed in 2000^4^. In this system, sulfide clusters in Chevrel-type Mo_6_S_8_ were used as cathodes, and a magnesium organohaloaluminate salt in tetrahydrofuran (THF) was used as the electrolyte. However, the energy density remained rather constrained by the cathode material, and the narrow potential window, corrosion, and safety problems posed by the electrolyte have hampered the commercial realization of these batteries. Recently, magnesium deposition and dissolution obtained by using magnesium bis(trifluoromethylsulfonyl)imide (Mg(TFSI)_2_) with glyme–diglyme have been reported^5^. The anodic stability of this electrolyte is higher than 3.0 V *vs*. Mg^2+^/Mg, and high-voltage cathode materials can be used in this electrolyte. Even though extensive research has been performed on cathode materials^6^, breakthroughs are awaited for the development of practically usable rechargeable magnesium batteries. In this study, we have attempted to address the problems related to cathode materials by using an ion-exchanged polyanion cathode (*i.e*., MgFeSiO_4_) and constructed a rechargeable magnesium battery using this high-energy-density cathode material.

The choice of cathode materials for magnesium battery is extremely limited because divalent Mg^2+^ insertion/extraction in host compounds is difficult, apparently due to the stronger ionic interaction and harder charge redistribution of magnesium compared to lithium ions^7^. For decades, various cathode materials have been proposed, including the molybdenum chalcogenides^4^,^7^, V_2_O_5_^8^, TiS_2_ nanotubes^9^, graphene-like MoS_2_^10^, todorokite-type/hollandite-type MnO_2_^11^,^12^, and sulfur^13^. Nevertheless, these candidates cannot achieve high capacity, high voltage, and excellent cyclability in parallel. Orthosilicates such as olivine-type Mg*M*SiO_4_ (*M* = Fe, Mn, Co) are another group of promising candidates, because the theoretical capacities of Mg*M*SiO_4_ exceed 300 mAh·g^−1^ and the operating voltages are expected to be higher than that of conventional magnesium battery cathode materials^14^. These silicate frameworks possess tetrahedral polyanions (SiO_4_^4−^) which are expected to afford lattice stabilization for magnesium (de)intercalation through the presence of strong Si–O bonds. Amongst this family of silicates, MgFeSiO_4_ is expected to be inexpensive because its constituent elements are abundant; in fact, the mineral olivine, (Mg, Fe)_2_SiO_4_ occurs naturally in significant amounts. The utilization of olivine-type MgFeSiO_4_ as a cathode material for rechargeable Mg batteries has previously been reported^15^. However, its innate electrochemical properties have not been adequately examined owing to the contribution of the electrochemical reaction from the copper current collector with the electrolyte, as reported recently^16^.

In olivine compounds such as LiFePO_4_, the lithium cation is located in a distorted octahedral site such that its diffusion pathway is one-dimensional (1D)^17^,^18^; in this case, the mobility of the carrier ions is easily impeded once defects are generated in the pathway (Fig. S1)^19^. The thermodynamically stable phase of MgFeSiO_4_ with the olivine-type structure possesses some degree of mixing between different octahedral Mg and Fe crystallographic sites^20^, and a similar limitation imposed by low dimensionality may occur. We therefore attempted to improve electrode kinetics by preparing a meta-stable phase of MgFeSiO_4_ via the electrochemical ion exchange of Li_2_FeSiO_4_, rather than by the conventional solid state synthesis. In Li_2_FeSiO_4_, the SiO_4_ tetrahedra are arranged in the same way as in the Mg*M*SiO_4_ olivine structure, but with slightly different spacings, so that Li and Fe are in tetrahedral rather than octahedral coordination, providing a 2D network (Fig. 1a, left).

The preparation of MgFeSiO_4_ involves, two electrochemical processes, namely, 2Li^+^ extraction from Li_2_FeSiO_4_ followed by Mg^2+^ insertion. Details regarding the characterization of as-prepared Li_2_FeSiO_4_ are shown in Supporting Information (Figs. S2, S3, S4 and Table S1). The complete extraction of Li^+^ from Li_2_FeSiO_4_ is performed in a Li-ion battery cell (as demonstrated previously^21^,^22^), and then Mg^2+^ is inserted into FeSiO_4_ after changing the electrolyte to one containing a Mg salt. The voltage profiles for the Li extraction and the Mg insertion processes are shown in Fig. 1b. To extract two Li^+^ from Li_2_FeSiO_4_, the cells are charged to the theoretical capacity of Li_2_FeSiO_4_ (*i.e*., 331 mAh·g^−1^) within an appropriate voltage range. The subsequent discharge processes in the Mg salt electrolyte deliver a capacity of approximately 330 mAh·g^−1^, demonstrating for the first time the ability to insert Mg^2+^ into FeSiO_4_. In this measurement, to improve electronic conduction path in the electrodes, an appreciable amount of carbon was used, which might cause side reactions without the contribution of the active materials. However, the composite electrode without the active materials exhibits little charge–discharge capacity (as shown in Fig. S5). Therefore, the obtained charge and discharge capacity emanates mainly from reaction of active materials.

Remarkably, MgFeSiO_4_ prepared via ion exchange undergoes reversible electrochemical charge-discharge processes. The charge-discharge profiles in a magnesium battery cell of the electrochemically prepared MgFeSiO_4_ are shown in Fig. 1c. The charge–discharge reaction was limited to within 330 mAh·g^−1^ to prevent the contribution of other reactions such as electrolyte decomposition. Considering the electrochemical window of magnesium (trifluoromethylsulfonyl)imide (Mg(TFSI)_2_) in acetonitrile (0.5 M), which is shown in Fig. S6 in Supporting Information, it can be concluded that the electrolyte was stable in our experiments. Although the charge-discharge potential appears slightly shifted, a reversible reaction is attainable with almost one Mg^2+^ insertion and extraction. The achieved discharge capacity of MgFeSiO_4_ is approximately twice that of conventional LIB cathodes such as LiCoO_2_ and LiFePO_4_. The average charge-discharge potential is −0.1 V *vs.* Ag^+^/Ag, which corresponds to 2.4 V *vs*. Mg^2+^/Mg according to the literature^23^. The energy density is estimated to be 746 Wh·kg^−1^, which far exceeds that of the Chevrel phases of Mo_6_S_8_ (~135 Wh·kg^−1^)^4^ and other cathode materials reported to date, such as V_2_O_5_ (~400 Wh·kg^−1^)^6^ and *α*-MnO_2_ (~560 Wh·kg^−1^)^11^.

Structural changes were investigated by X-ray diffraction using a synchrotron source. Rietveld refinement of Li_2_FeSiO_4_ (Fig. S2 and Table S1) resulted in a *P*2_1_/*n* monoclinic structure comprising of the 2D network of SiO_4_ and FeO_4_ tetrahedra, which agrees well with the literature^24^. It is predicted that Li_2–*x*_FeSiO_4_ has various polymorphs with 2D and 3D networks upon electrochemical reaction^25^. Indeed, our recent XRD study demonstrated that the initial 2D monoclinic (*P*2_1_/*n*) structure in Li_2_FeSiO_4_ is converted to a 3D orthorhombic (*Pnma*) structure in LiFeSiO_4_^26^. In this study, we performed further delithiation from LiFeSiO_4_ and found that the *Pnma* orthorhombic 3D structure is retained in FeSiO_4_ (Figs. S7 and S8 and Tables S2 and S3 in Supporting Information). Namely, the 2D network in Li_2_FeSiO_4_ (Fig. 1a, left) is transformed to a 3D network in FeSiO_4_ (Fig. 1a, center). Note that satisfactory refinement could not be obtained when structural models with the original Li_2_FeSiO_4_-type 2D network were used.

After the subsequent Mg^2+^ insertion and extraction processes, the XRD patterns reversibly change (Fig. S9), while the orthorhombic crystal structure is maintained as shown in Table 1. The lattice parameters of Mg_1–*x*_FeSiO_4_ appear to follow the Vegard's law. Although refinement of MgFeSiO_4_ and Mg_0.5_FeSiO_4_ was difficult because of poor data quality, these observations strongly indicate that the 3D network in FeSiO_4_ is retained upon the Mg^2+^ insertion/extraction (Fig. 1a, right). The cell volume reduction by Mg^2+^ insertion can be explained by the reduced repulsion between Fe and Si cations (Fe^2+^-Si^4+^ in MgFeSiO_4_ *vs*. Fe^4+^-Si^4+^ in FeSiO_4_), consistent with recent theoretical reports^14^. Such a 3D network can be beneficial in terms of the stability of cathode materials upon Mg^2+^ insertion/extraction.

The full de-intercalation of Li_2_FeSiO_4_ or MgFeSiO_4_ would presumably result in Fe^4+^, therefore the charge compensation mechanism was investigated using Fe *K*-edge X-ray absorption near edge structure (XANES), as shown in Fig. 2a. The higher and lower energy shifts of the XANES spectra at the Fe *K*-edge correspond, respectively, to increase and decrease in the oxidation state of the Fe ions, as confirmed in previous iron silicate systems^22^,^27^. During the lithium extraction process, a significant shift is observed from Li_2_FeSiO_4_ to LiFeSiO_4_. This corresponds to the oxidation of divalent Fe ions to the trivalent state, which is dominated by the outermost orbital of the Fe-3*d* band. Between LiFeSiO_4_ and FeSiO_4_, only a small shift is observed, as has also been noted by others upon a more than one lithium extraction from Li_2_FeSiO_4_^22^. The small edge shift suggests that other charge compensation mechanisms should be occurring between LiFeSiO_4_ and FeSiO_4_. In the subsequent Mg^2+^ (de)insertion processes, reversible shifts in the absorption energies are observed as shown in Fig. 2b. While the energy shift in the XANES spectrum is small from FeSiO_4_ to Mg_0.5_FeSiO_4_ (similar to what was observed in the LiFeSiO_4_–FeSiO_4_ regime), a significant shift is observed from Mg_0.5_FeSiO_4_ to MgFeSiO_4_. A similar trend was also observed during the magnesium extraction process, as is apparent in Fig. 2c.

O *K*-edge X-ray absorption spectroscopy (XAS) measurements give further insight for FeSiO_4_ (Fig. 2d). The pre-edge peak intensity at 529 eV increases as Fe is oxidized; this indicates the degree of hybridization between the Fe 3*d* states and O 2*p* states^28^. Such a strong hybridization between the metal and ligand can lead to creation of ligand holes, suggesting this process as the redox mechanism during the charge-discharge reaction of ion-exchanged MgFeSiO_4_. In general, anion redox processes can contribute to large capacities, as has recently been revealed in the lithium (de)intercalation of Li_2_(Ru,Sn)O_3_^29^. The charge compensation process dominantly occurs within the Fe 3*d* orbital in the Mg_0.5_FeSiO_4_–FeSiO_4_ regime. Conversely, the O 2*p* orbital plays an important role in the oxidation/reduction process between the Mg_0.5_FeSiO_4_ and FeSiO_4_ regimes by creating holes at ligand sites. Such different electronic structural changes influence the charge–discharge potential profiles (shown in Fig. 1c), in which two-stage sloping profiles are observed. The theoretical potential based on DFT calculations carried out using the experimental XRD data (shown in Fig. S7 and S8 in Supporting Information) is in good agreement, particularly between the MgFeSiO_4_ and Mg_0.5_FeSiO_4_ regimes. However, there is a discrepancy between the experimental and theoretical potential values in the Mg_0.5_FeSiO_4_–FeSiO_4_ regime, where the contribution of O 2*p* orbital states is more dominant. This discrepancy should not come as a surprise, considering that DFT calculations in this study do not fully take into account the exchange–correlation interaction for oxygen states.

For a working magnesium battery based on ion-exchanged MgFeSiO_4_, further improvement in the electrolyte is pivotal because magnesium deposition/dissolution cannot be achieved using (Mg(TFSI)_2_) in acetonitrile as a solvent. Previously proposed electrolytes such as magnesium organohaloaluminates and hexamethyldisilazide magnesium chloride in THF^13^,^23^ have narrow potential windows due to the use of chloride and THF. Additionally, these electrolytes suffer from corrosion (due to the halide) and also pose safety problems with regard to flammability (due to the volatility of THF). An alternative electrolyte, employing a different magnesium salt dissolved in a less volatile solvent, would therefore be desirable. Here, we present our results using magnesium bis(trifluoromethylsulfonyl)imide (Mg(TFSI)_2_) dissolved in triglyme. Cyclic voltammograms of a platinum working electrode in the Mg(TFSI)_2_–triglyme electrolyte at 100°C are shown in Fig. 3a. The cathodic and anodic peaks correspond to magnesium deposition and dissolution, respectively. The anodic stability in this electrolyte is higher than 3.5 V *vs*. Mg^2+^/Mg. This value is higher than that of the wide potential window of organohaloaluminates, *etc*., in THF, recently reported by Muldoon and co-authors^30^. The deposited product was characterized by XRD and scanning electron microscopy (SEM) (Figs. 3b and 3c). The diffraction pattern of the deposited product is fully indexed to the *P*6_3_/*mmc* space group, consistent with the formation of Mg metal. Particles approximately 5 μm thick were deposited on the platinum working electrode without dendrite formation. Most recently, magnesium deposition and dissolution obtained using Mg(TFSI)_2_ with glyme–diglyme as a solvent have been reported^5^. The boiling point of triglyme is higher than that of the glyme–diglyme solvent, which is favorable for stable battery operation under various temperatures. Our results validate that the Mg(TFSI)_2_–triglyme system, in addition to the reported glyme–diglyme system^5^, can be used in rechargeable magnesium batteries in combination with high-voltage cathode materials.

As a proof-of-concept, we therefore propose a novel rechargeable magnesium battery system as shown in Fig. 4a, where ion-exchanged MgFeSiO_4_ and Mg metal are used as the cathode and anode, respectively, and Mg(TFSI)_2_–triglyme as the electrolyte. These materials would be highly beneficial for increasing the energy density of the electrode materials in magnesium batteries without imposing significant constraints on available resources. The chemical and thermal stabilities afforded by the polyanion moieties are also suitable for cathode materials. The Mg(TFSI)_2_–triglyme electrolyte does not contain Cl-, Br- or THF-based solvents. This enables the safe operation of rechargeable magnesium batteries without corrosion and low flammability. Using ion-exchanged MgFeSiO_4_ and Mg(TFSI)_2_–triglyme, charge–discharge measurements for the magnesium rechargeable battery full cell were performed at 100°C; the obtained results are shown in Fig. 4b. A reversible charge–discharge capacity of 166 mAh·g^−1^ was obtained, which was calculated on the basis of the mass of the active material (*viz*., ion-exchanged MgFeSiO_4_ cathode), despite the effect of anode polarization on this profile. When Mg(TFSI)_2_–triglyme is used as the electrolyte, only half of the theoretical capacity of the cathode material could be obtained owing to the high polarization and elevated temperature operation needed (Fig. S11). The high polarization might presumably be arising from the low Mg^2+^ cation flux in the triglyme solvent. In a Li(TFSI)–triglyme system, high polarization was also reported^31^. Further improvements in the electrolyte and morphology control of the composite electrodes are essential.

This study demonstrates ion-exchanged MgFeSiO_4_ as a feasible cathode material for use in rechargeable magnesium batteries. The application of ion-exchanged MgFeSiO_4_ polyanion compounds as rechargeable magnesium battery cathode materials provides a capacity of more than 300 mAh·g^−1^ at an average potential of 2.4 V *vs*. Mg^2+^/Mg, with good retention upon cycling. The electronic and crystal structure of ion-exchanged MgFeSiO_4_ changes during the charging and discharging processes, which demonstrates the (de)insertion of magnesium in the host structure. Batteries using a combination of ion-exchanged MgFeSiO_4_ and the Mg(TFSI)_2_–triglyme electrolyte represent a prototype for a low-cost, high-energy-density rechargeable magnesium battery in which no toxic or explosive components are used.

## Methods

### Material synthesis

Cathode materials were synthesized using a solid-state reaction. Amorphous SiO_2_ (99.9%), FeC_2_O_4_·2H_2_O (99%), and Li_2_CO_3_ (99%) powders were weighed in a molar ratio of 1:1:1. The powders were mixed using a ball mill in acetone. 10 *wt*% Ketjen carbon black was added to improve the electronic conductivity. Mixing was performed using a plenary ball mill (Fritsch LP-6) at 400 rpm for 6 h. The obtained slurry was dried at room temperature under vacuum. The dried powder was pelletized and calcined at 800°C for 6 h under Ar flow. The obtained powder was thereafter transferred to an argon-filled glove box, owing to the inherent sensitivity of the material upon air exposure.

### Materials characterization

The products were characterized by X-ray diffraction (XRD), scanning electron microscopy (SEM), and transmission electron microscopy (TEM). Conventional XRD measurements were performed at room temperature on a Rigaku Rint-2200 diffractometer using Cu *K*α radiation. The diffraction data were collected in a 2*θ* range of 10° to 60° with a step size of 0.04°. SEM micrographs were recorded with a JSM-890 (JEOL) operated at 15 kV. TEM measurements were performed with an HR-9000 (Hitachi) operated at 200 kV, taking care not to expose the samples to air.

### Electrochemical measurements

Electrodes were prepared from the basic active material (Li_2_FeSiO_4_), to which carbon (acetylene black) was added, and ball-milled at 400 rpm for 30 minutes. Polytetrafluoroethylene (PTFE) binder was thereafter added to obtain a final weight ratio of 40:50:10. Two-electrode cells were prepared using metallic lithium as the counter electrode. The electrolyte was 1 M LiClO_4_ in propylene carbonate. Cells were charged at 55°C to a capacity commensurate with the extraction of two Li^+^ (theoretical capacity of *ca.* 331 mAh·g^−1^). After the charge processes, the cells were dismantled in an Ar-filled glove box. The working electrode was rinsed several times with dimethyl carbonate and dried in vacuum. The dried active electrodes were pressed on Pt mesh as the working electrode. Three-electrode cells were used for Mg battery measurements. The electrolyte consisted of 0.5 M magnesium (trifluoromethylsulfonyl)imide (Mg(TFSI)_2_) in acetonitrile. A Mg ribbon was used as the anode. As the reference electrode, a silver wire was inserted into a solution of 0.01 M AgNO_3_ and 0.1 M Mg(TFSI)_2_ in acetonitrile. This solution, contained in an additional glass tube, was brought into contact with the Mg(TFSI)_2_/acetonitrile solution via a microporous glass membrane. The galvanostatic charge–discharge measurements were carried out at 55°C. The cells were cycled at a C/50 rate within the capacity ranges of one Mg^2+^ per Fe. The upper and lower cut-off potentials were set at 1.0 V *vs.* Ag^+^/Ag and −1.0 V *vs*. Ag^+^/Ag, respectively.

### Mg deposition and dissolution test

Three-electrode cells were used. A platinum sheet and a Mg rod were used as the working and the counter electrodes, respectively. Mg(TFSI)_2_/triglyme (1:5 molar ratio) was used as the electrolyte. As a reference electrode, a silver wire was inserted into a solution of 0.01 M AgNO_3_ and 0.1 M Mg(TFSI)_2_ in triglyme. Cyclic voltammetry was performed in the potential range of −3.75–1.5 V at a potential sweep rate of 1.0 mV s^−1^ at 100°C.

### Mg rechargeable battery test

Two-electrode cells were used. Ion-exchanged MgFeSiO_4_ and Mg metal were used as the cathode and anode, respectively. The galvanostatic charge–discharge measurements were performed at a C/50 rate at 100°C. The capacity range was limited to 0.5 Mg^2+^ per Fe. The upper and lower cut-off voltages were set at 1.0 V *vs.* Mg^2+^/Mg and −1.0 V *vs.* Mg^2+^/Mg, respectively.

### XRD measurements

After the electrochemical measurements, the charged/discharged electrodes were rinsed several times with acetonitrile. Powder samples were then loaded into a glass capillary and sealed in an argon-filled glove box to eliminate the exposure of the samples to air. Synchrotron X-ray diffraction patterns were collected at the beam line BL02B2 of SPring-8, Japan, equipped with a large Debye-Scherer camera. The wavelength of the incident X-ray beam was set to 0.5 Å using a double-crystal silicon (111) monochromator, which was calibrated with a CeO_2_ standard. Full pattern matching and Rietveld refinements were performed with the JANA2006 program package. The backgrounds were interpolated by a Chebyshev function, and the peak shapes were described by a pseudo-Voigt function.

### XAS measurements

For the Fe *K*-edge XAS measurements, charged/discharged Mg_1–*x*_FeSiO_4_ electrodes were intimately mixed with boron nitride powder and pressed into pellets. The pellets were sealed in laminated packets in an argon-filled glove box. The XAS spectra were measured in the energy region of the Fe *K*-edge at room temperature in transmission mode at the beam line of the SPring-8 synchrotron radiation facility (BL01B1 and BL14B2) in Hyogo, Japan. Treatment of the raw X-ray absorption data was performed with the Athena package, allowing for alignment and normalization. As for the O *K*-edge XAS measurements, the electrodes were transferred to the measurement chamber without exposing the samples to air. The spectra were measured at BL-2 of the SR center at Ritsumeikan University (Japan). The spectra were collected in fluorescence yield mode.

## Author Contributions

Y.O. and Y.U. conceived the experiments. T.Ma. and Z.H. synthesized the materials. T.A. designed the electrolyte. T.Mo. and K.Y. performed Mg deposition and dissolution measurements. M.H., T.O. and T. Ma. performed charge and discharge measurements. C.T., Y.K. and H.K. analyzed the crystal structure of the electrodes. J.K. and T.Mi. conducted synchrotron XRD measurements. Y.O. and T.Ma. analyzed the XAS data. Y.O. and T.Ma. wrote the manuscript. Y.K. performed the DFT calculation. All authors discussed the results and contributed to the final version of the manuscript.

## Supplementary Material

Supplementary Information Supplementaly information

## Figures and Tables

**Figure 1:**
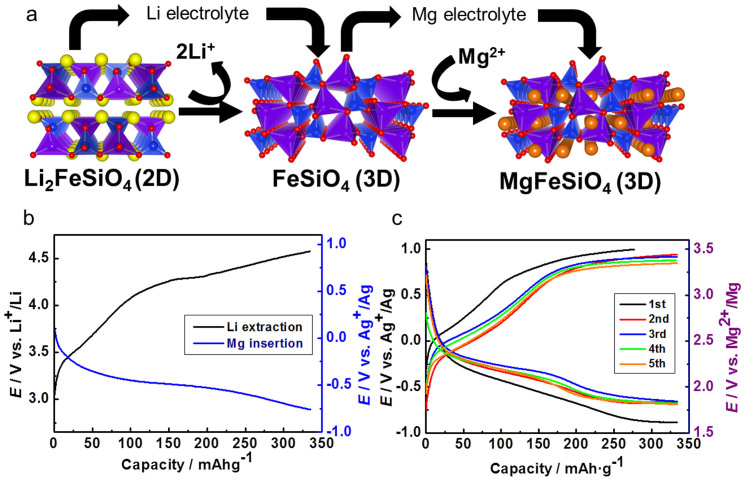
Preparation of ion-exchanged MgFeSiO_4_ and charge-discharge profiles. (a) Schematic illustration of the ion-exchange methodology for the electrochemical synthesis of MgFeSiO_4_ from Li_2_FeSiO_4_. Two-dimensional (2D) framework of Li_2_FeSiO_4_ and three-dimensional (3D) framework of FeSiO_4_ and MgFeSiO_4_. The 3D framework can incorporate Mg ions in the interspace (void). (b) Charge–discharge profiles for ion exchange process from Li_2_FeSiO_4_ to MgFeSiO_4_. For Li extraction process, two-electrode cells using lithium as counter electrodes were used. Electrolyte was 1 M LiClO_4_ in propylene carbonate. For Mg insertion process, three-electrode cells (using Mg metal counter electrode and silver reference electrode) were used. Electrolyte was 0.5 M magnesium (trifluoromethylsulfonyl)imide (Mg(TFSI)_2_) in acetonitrile as solvent. Measurement temperature was 55°C. Current density was 6.62 mA·g^−1^ (Li_2_FeSiO_4_). (c) Charge–discharge profiles of ion-exchanged MgFeSiO_4_. Three-electrode cells using Mg metal counter electrode and silver reference electrode were used. Electrolyte was 0.5 M magnesium (trifluoromethylsulfonyl)imide (Mg(TFSI)_2_) in acetonitrile (solvent). Measurement temperature was 55°C. Current density was 6.62 mA·g^−1^ (MgFeSiO_4_).

**Figure 2:**
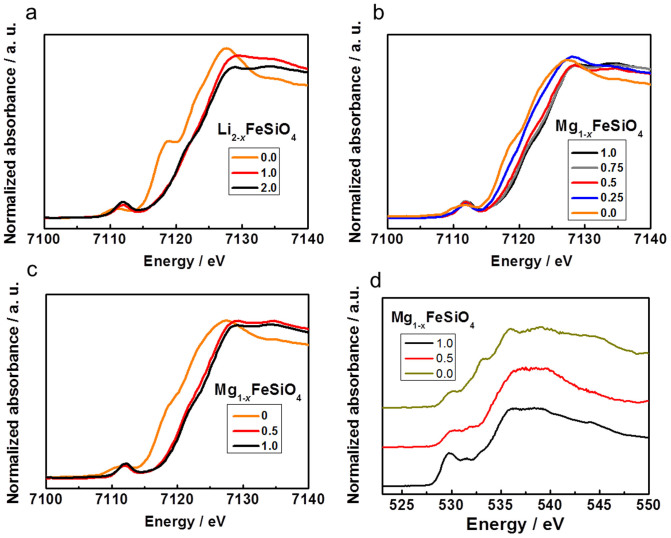
Characterization of charged and discharged Mg_1–*x*_FeSiO_4_ electrodes. (a) X-ray absorption near edge structure (XANES) spectra at the Fe *K*-edge of Li_2–*x*_FeSiO_4_ during the initial charge (Li^+^ extraction) with a Li electrolyte. (b) XANES spectra of Mg_1–*x*_FeSiO_4_ during the initial discharge and (c) the initial charge using a Mg electrolyte during Mg^2+^ insertion and extraction, respectively. (d) O *K*-edge XAS spectra of Mg_1–*x*_FeSiO_4_ electrode during the initial Mg^2+^ insertion process.

**Figure 3:**
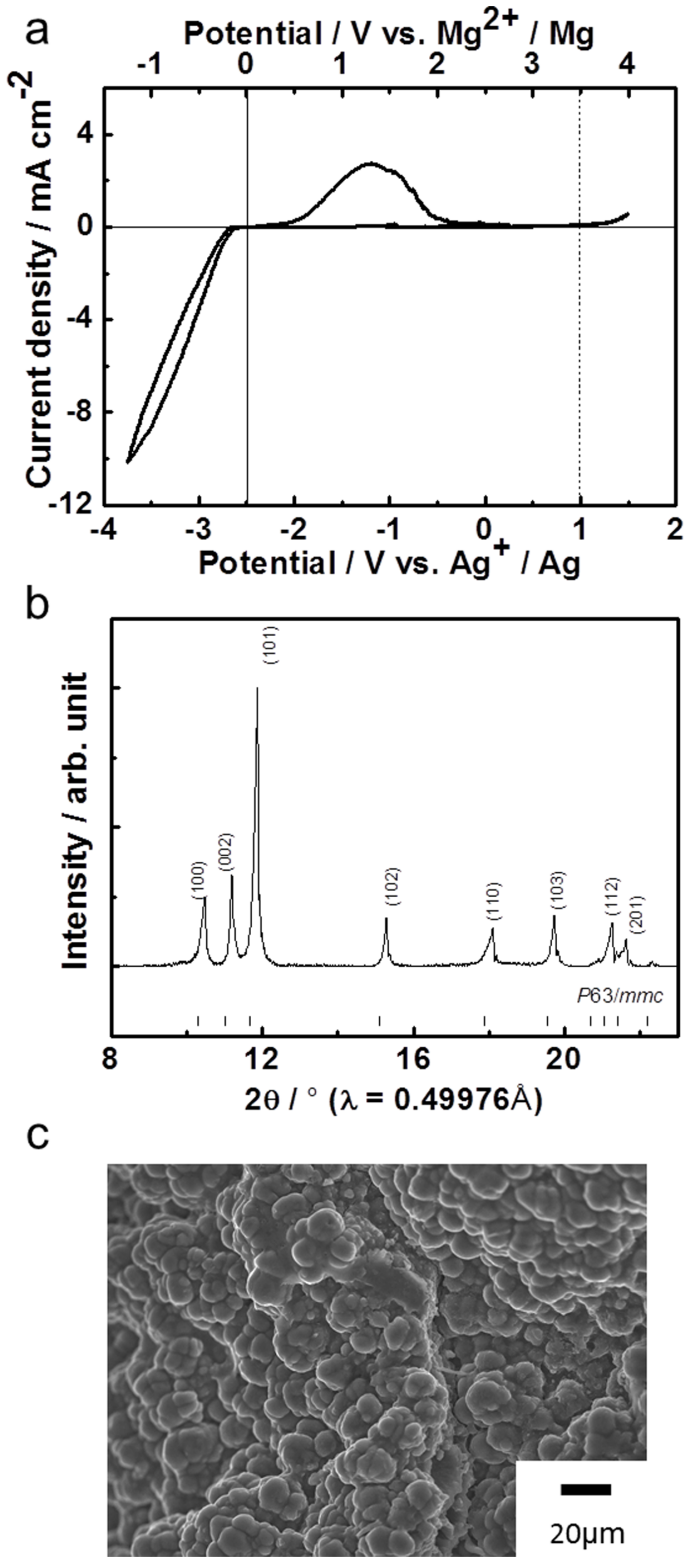
Mg deposition and dissolution in the Mg(TFSI)_2_–triglyme electrolyte. (a) Cyclic voltammograms of platinum electrode in Mg(TFSI)_2_/triglyme (1:5 molar ratio). Three-electrode cells using Mg metal counter electrode and silver reference electrode were used. Potential sweep rate was set at 1.0 mV s^−1^, and measurements were conducted at 100°C. (b) XRD pattern of the deposited products. (c) SEM image of the deposited products.

**Figure 4:**
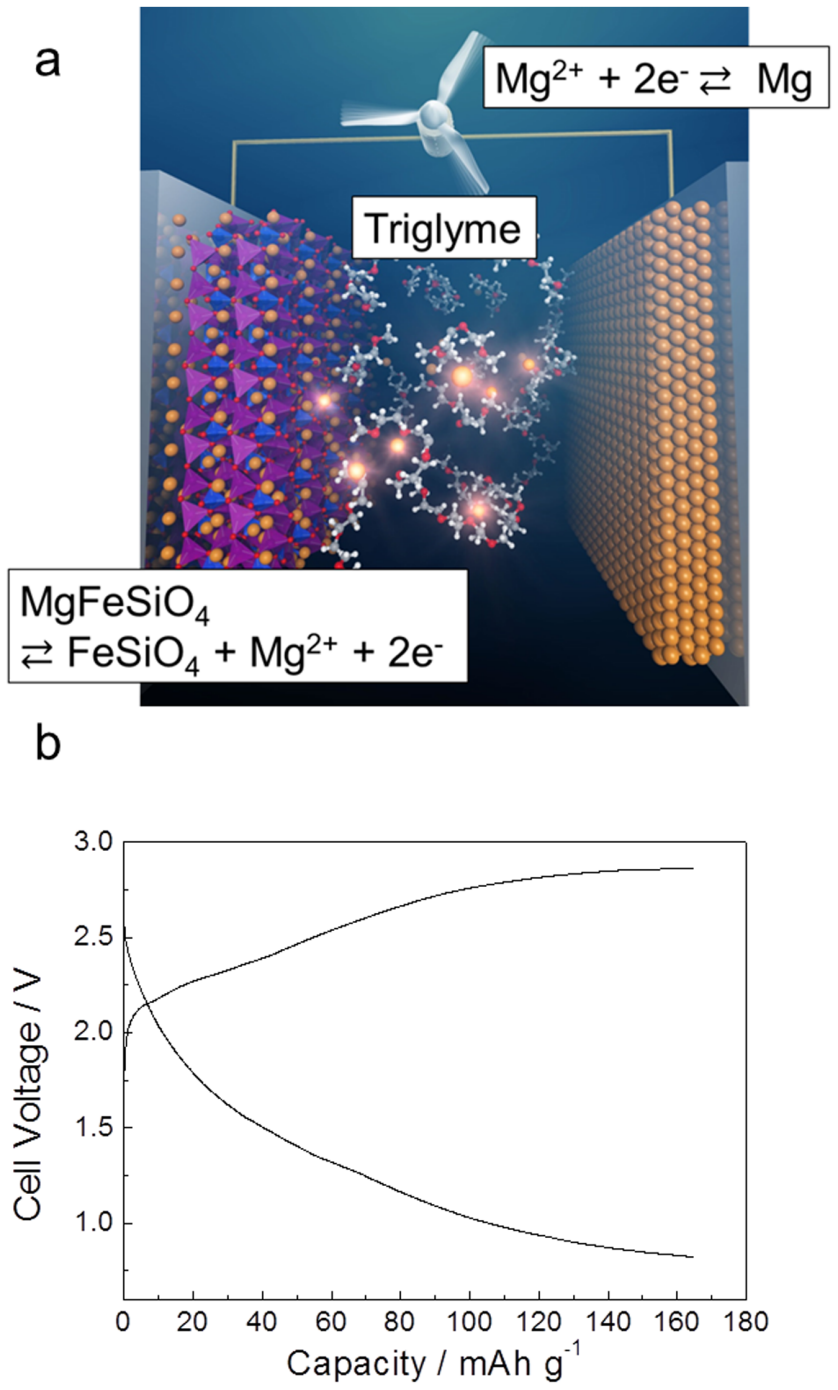
Prototype of a high energy-density rechargeable Mg battery. (a) Schematic illustration of the proposed Mg battery system. MgFeSiO_4_ and Mg metal are used as the cathode and the anode, respectively. Mg(TFSI)_2_–triglyme is used as the electrolyte. (b) Charge–discharge voltage profiles of proposed Mg rechargeable battery. Two-electrode cells with ion-exchanged MgFeSiO_4_ (cathode) and Mg (anode) were used. Measurements were performed at 100°C at a current density of 6.62 mA·g^−1^. Capacity range was limited to 0.5 Mg^2+^ per Fe.

**Table 1:** Lattice parameters and cell volumes for Li_2_FeSiO_4_ and Mg_1–*x*_FeSiO_4_ during magnesium insertion and extraction

	Lattice	*a* (Å)	*b* (Å)	*c* (Å)	V (Å^3^)
*Li_2_FeSiO_4_ (as-prepared)	Monoclinic	8.2433(4)	5.0226(1)	8.2373(3)	336.31
*FeSiO_4_ (delithiated)	Orthorhombic	10.3969(20)	6.5618(16)	5.0334(8)	343.39
†Mg_0.5_FeSiO_4_ (magnesiated)	Orthorhombic	10.2829(6)	6.5767(5)	5.0019(3)	338.27
†MgFeSiO_4_ (magnesiated)	Orthorhombic	10.2464(21)	6.5038(12)	4.9427(9)	329.38
†Mg_0.5_FeSiO_4_ (demagnesiated)	Orthorhombic	10.2526(7)	6.5582(7)	4.9985(3)	335.42
*FeSiO_4_ (demagnesiated)	Orthorhombic	10.3434(19)	6.5779(13)	5.0185(8)	341.45

*Values from Rietveld refinement of powder XRD data.

^†^Values from indexing powder XRD data.
