# Supplementary material for: High energy density rechargeable magnesium battery using earth-abundant and non-toxic elements
Source: Sci Rep. 2014 Jul 11;4:5622. doi: 10.1038/srep05622 (PMC4092329; doi:10.1038/srep05622)
Supplement: Supplementary Information — Supplementaly information [file srep05622-s1.pdf]

**Supporting information**

# High energy density rechargeable magnesium battery using earth-abundant and non-toxic elements

Yuki Orikasa[1*], Titus Masese[1], Yukinori Koyama[2], Takuya Mori[1], Masashi Hattori[1], Kentaro Yamamoto[1], Tetsuya Okado[1], Zhen-Dong Huang[1], Taketoshi Minato[2], Cédric Tassel[3, 4], Jungeun Kim[5], Yoji Kobayashi[3], Takeshi Abe[3], Hiroshi Kageyama[3, 6], Yoshiharu Uchimoto[1]

[1]Graduate School of Human and Environmental Studies, Kyoto University, Yoshida-nihonmatsu-cho, Sakyo-ku, Kyoto 606-8501, JAPAN

[2]Office of Society-Academia Collaboration for Innovation, Kyoto University, Gokasho, Uji, Kyoto 611-0011, JAPAN

[3]Graduate School of Engineering, Kyoto University, Katsura-cho, Nishikyo-ku, Kyoto 615-8510, JAPAN

[4]The Hakubi Center for Advanced Research, Kyoto University, Yoshida-Ushinomiya-cho, Sakyo-ku, Kyoto 606-8302, JAPAN

[5]Japan Synchrotron Radiation Research Institute, 1-1-1 Kouto, Sayo-cho, Sayo-gun, Hyogo 679-5198, JAPAN

[6]Institute for Integrated Cell-Material Sciences, Kyoto University, Yoshida-Ushinomiya-cho, Sakyo-ku, Kyoto 606-8302, JAPAN

*Correspondence to: Yuki Orikasa (orikasa.yuuki.2a@kyoto-u.ac.jp)

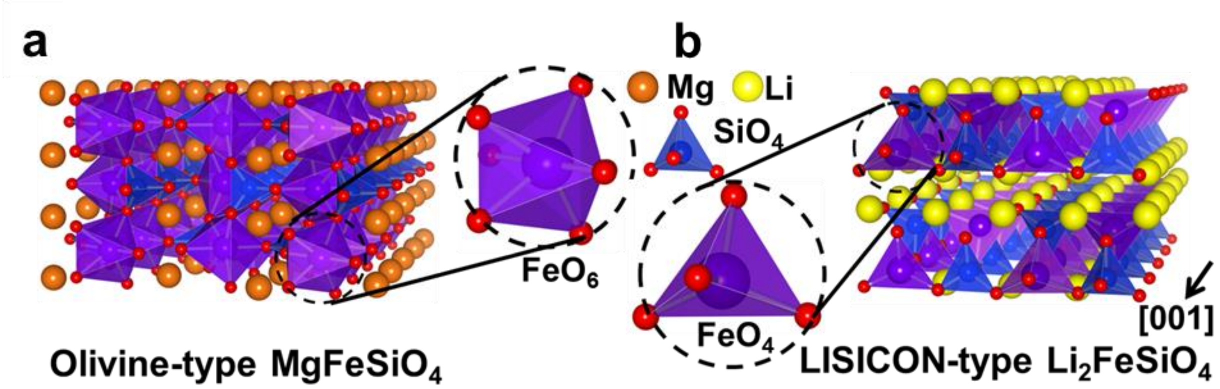


Fig. S1. Crystal structure showing (a) octahedral coordination in olivine-type $MgFeSiO_4$ and (b) tetrahedral coordination in as-prepared $Li_2FeSiO_4$.

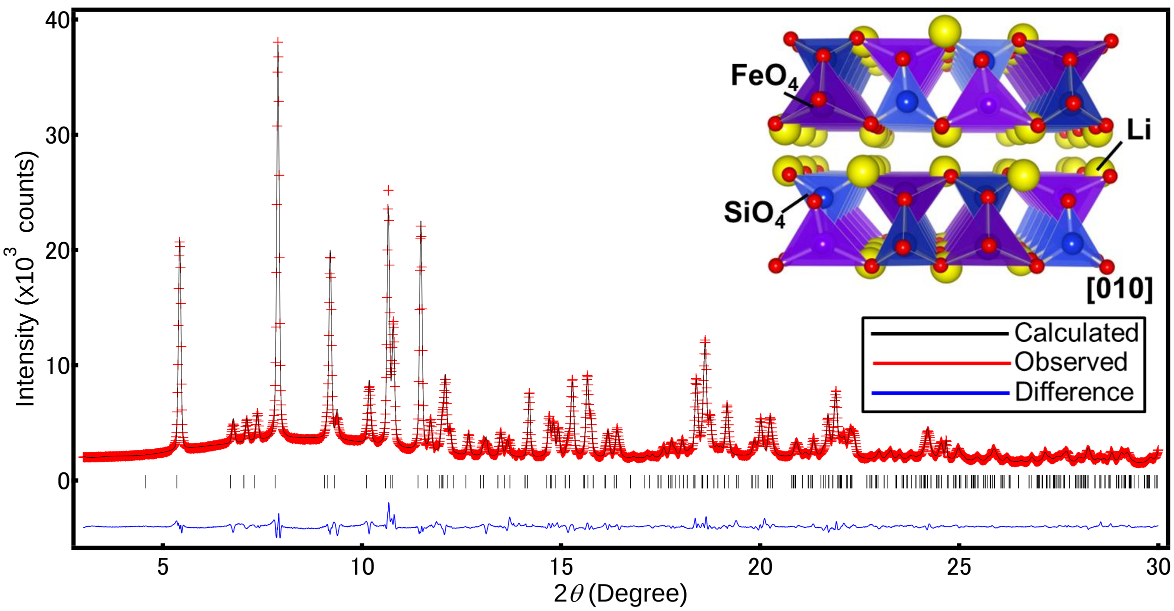


Fig. S2. Synchrotron XRD patterns (3° < $2\theta$ < 30° range) for pristine $Li_2FeSiO_4$. The wavelength used was 0.50005 Å. The refined crystal structure is also shown.

Table S1. Atomic coordinates (*x*, *y*, *z*), occupancies (*g*), and atomic displacement parameters (*U*) obtained by Rietveld refinement of synchrotron X-ray diffraction data for pristine $Li_2FeSiO_4$ in space group $P2_1/n$ (monoclinic) with lattice constants $a$ = 8.2433(4) Å, $b$ = 5.0226(1) Å, $c$ = 8.2373(3) Å, and $\beta$ = 99.09(0)°. The atomic position of Li and atomic displacement parameters of atoms were fixed according to the literature values (*1*).

| Atom | *g* | *x* | *y* | *z* | $100 \cdot U_{iso}$ |
|---|---|---|---|---|---|
| Li1 | 1.0 | 0.6637 | 0.8144 | 0.6811 | 0.93 |
| Li2 | 1.0 | 0.5913 | 0.2140 | 0.0768 | 0.95 |
| Fe1 | 0.9835(21) | 0.2907(3) | 0.7916(3) | 0.5408(4) | 0.83 |
| Si1 | 1.0 | 0.0452(5) | 0.8045(5) | 0.7916(5) | 0.70 |
| O1 | 1.0 | 0.8544(11) | 0.7077(15) | 0.8749(8) | 0.16 |
| O2 | 1.0 | 0.4212(9) | 0.2177(9) | 0.8749(8) | 0.84 |
| O3 | 1.0 | 0.7122(9) | 0.7629(8) | 0.4317(7) | 0.93 |
| O4 | 1.0 | 0.9699(9) | 0.8448(8) | 0.2142(9) | 0.96 |

$R_{wp}$=3.69%   $R_p$=2.57%   $\chi^2$=1.77

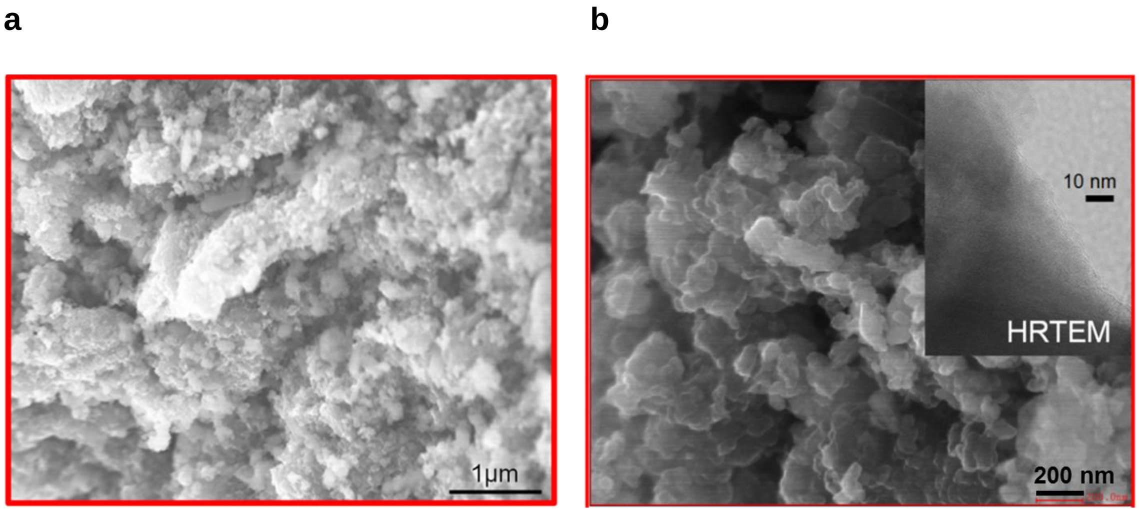


Fig. S3. (a) SEM image and (b) High resolution SEM image of the pristine $Li_2FeSiO_4$ nanoparticles. Inset shows HRTEM image of the pristine $Li_2FeSiO_4$ with an amorphous carbon coating. Typical particle size used in our study was *ca.* 50 nm.

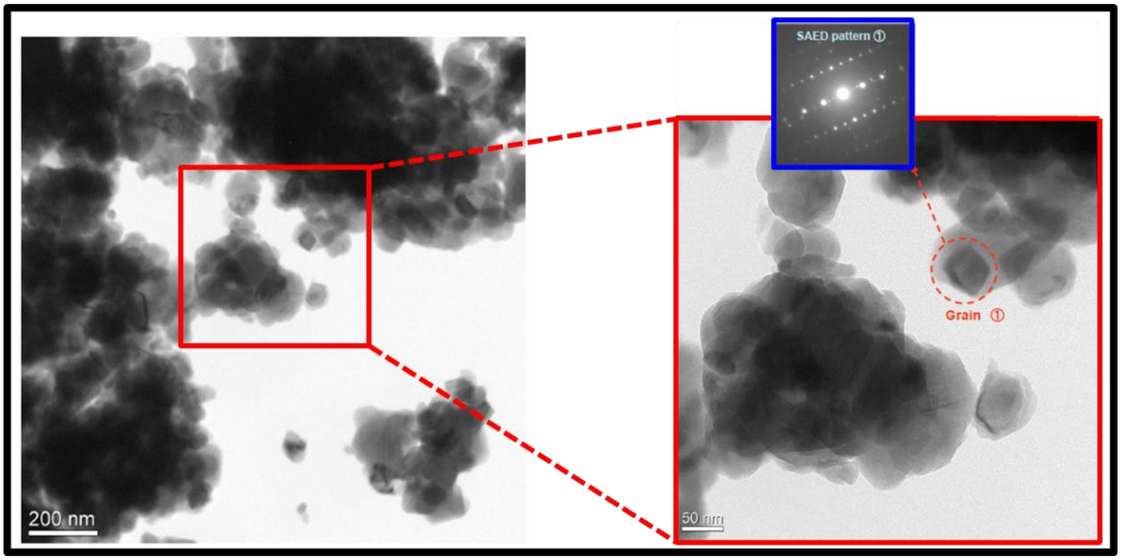


Fig. S4. TEM image coupled with high resolution TEM-SAED image of the pristine $Li_2FeSiO_4$ nanoparticle, revealing an array of pseudo-hexagonal symmetry dots that could be indexed, using the monoclinic ($P2_1/n$ space group) $Li_2FeSiO_4$ settings, as the [141] zone axis.

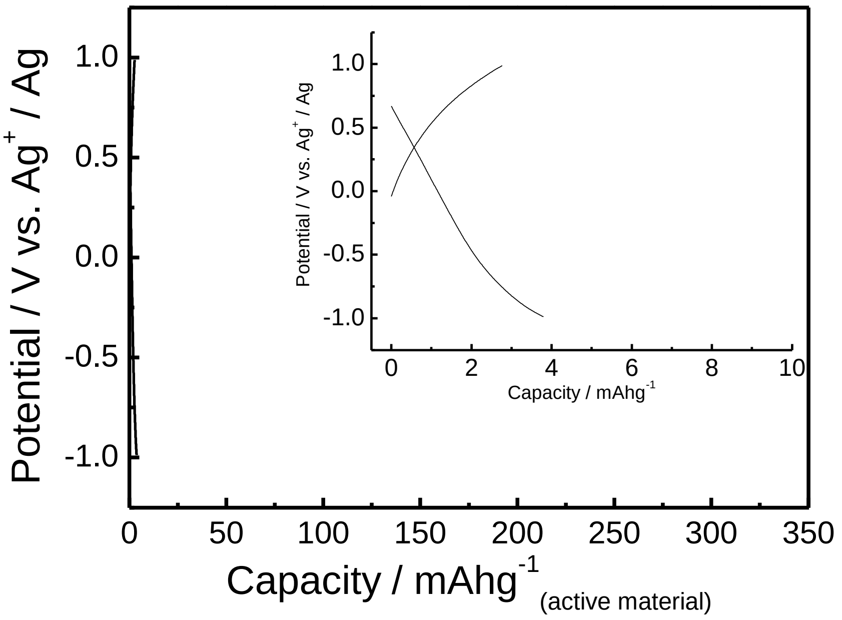


Fig. S5. Contribution of carbon (acetylene black) and PTFE to charge-discharge capacity. Acetylene black and PTFE binder was mixed in a weight ratio of 50: 10. The electrodes were pressed on Pt mesh as the working electrode. Three-electrode cells were used. The electrolyte consisted of 0.5 M magnesium (trifluoromethylsulfonyl)imide ($Mg(TFSI)_2$) in acetonitrile. A Mg ribbon was used as the anode. As the reference electrode, a silver wire was inserted into a solution of 0.01 M $AgNO_3$ and 0.1 M $Mg(TFSI)_2$ in acetonitrile. The galvanostatic charge and discharge measurements were carried out at 55°C. The current density was set to the charge / discharge current with C/50 rate supposing the composite electrode of $MgFeSiO_4$ / carbon / PTFE (40: 50: 10 *wt* %). The charge / discharge capacity from carbon and PTFE electrode is negligible. Therefore, the observed charge and discharge capacity arises from reaction of active materials.

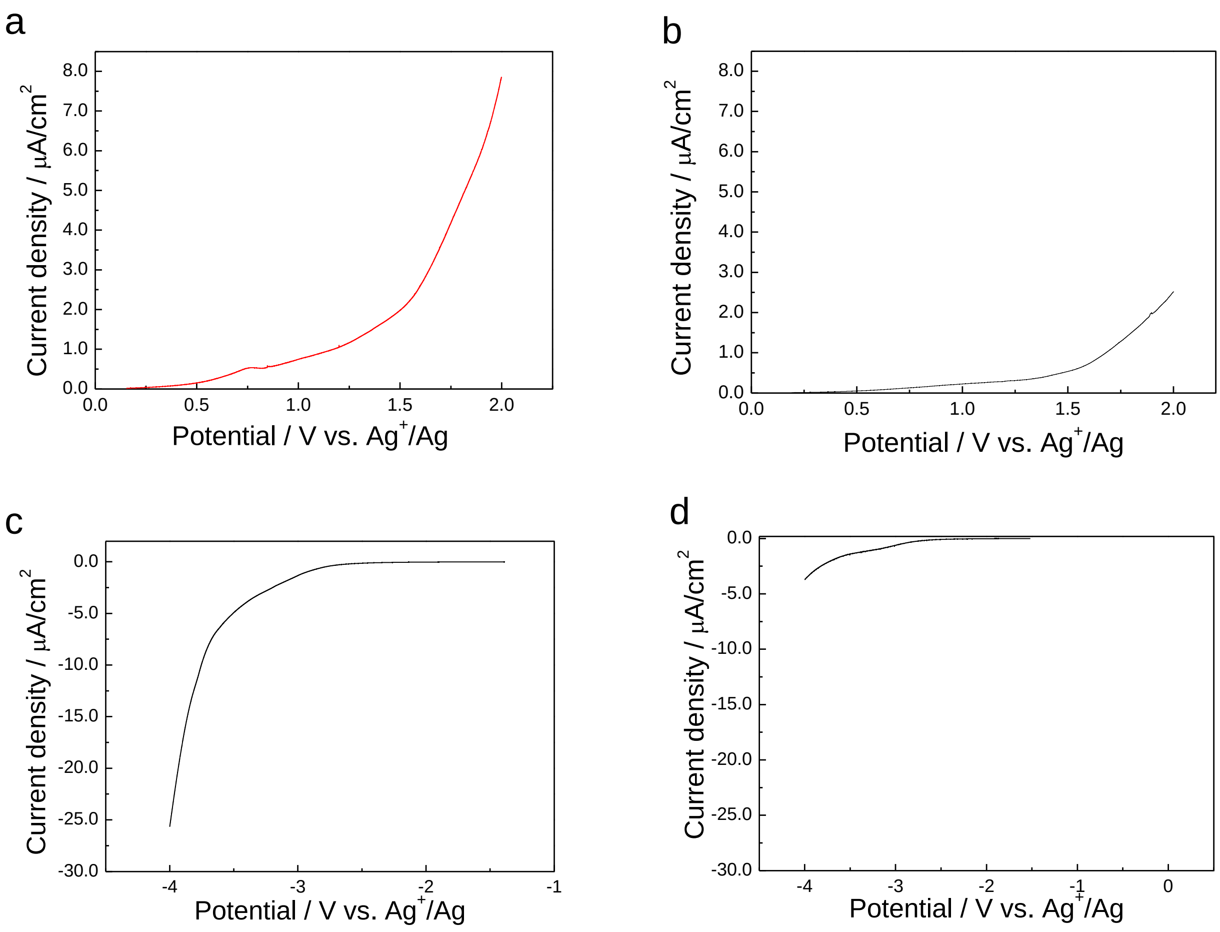


Fig. S6. Linear sweep voltammetry. Three-electrode cells using Pt working electrode, Mg metal counter electrode, and silver reference electrode were used. Electrolyte was 0.5 M magnesium (trifluoromethylsulfonyl)imide ($Mg(TFSI)_2$) in acetonitrile (solvent). Potential sweep rate was set at 1.0 mV $s^{-1}$, and measurements were conducted at 55°C (for (a) and (c)) and 25°C (for (b) and (d)).

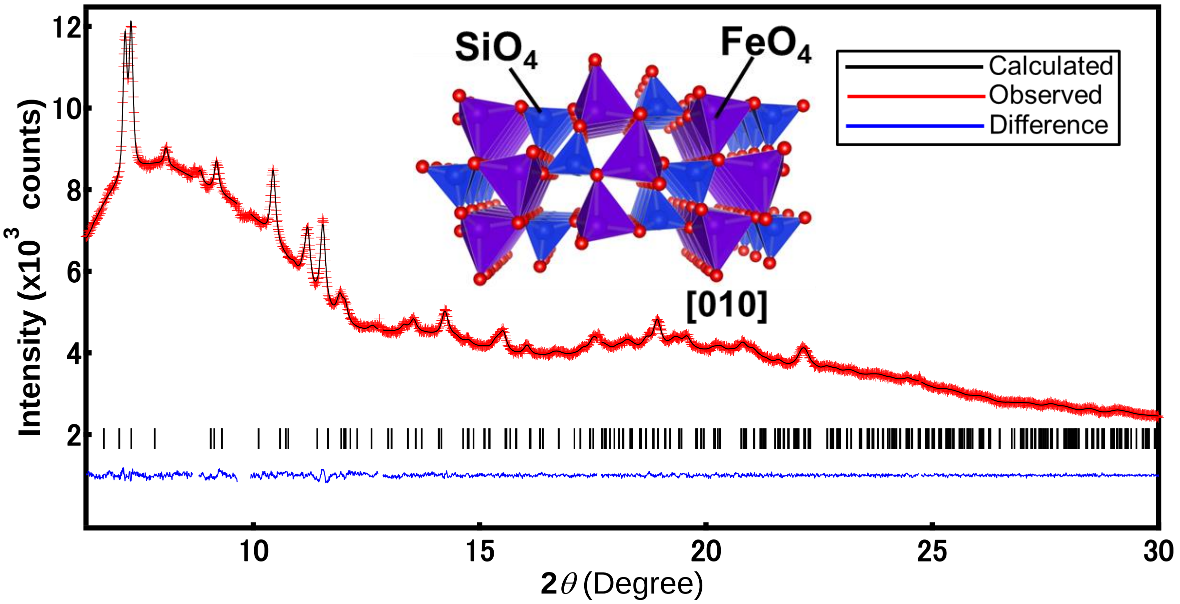


Fig. S7. Rietveld refinement pattern of synchrotron XRD data for electrochemically delithiated $FeSiO_4$ from $Li_2FeSiO_4$. Peaks that could not be properly indexed to the orthorhombic cell symmetry were excluded from the refinement. The refined orthorhombic 3D crystal structure of $FeSiO_4$ is also shown. Refinement using an orthorhombic 2D structural model $FeSiO_4$ gave high reliability values and the calculated bond valence summation of Si was unrealistic. An orthorhombic 3D structural model, however, yielded low reliability values and realistic summations of the bond valence to warrant the orthorhombic 3D framework as an appropriate model.

Table S2. Atomic coordinates, occupancies, and atomic displacement parameters obtained by Rietveld refinement of synchrotron XRD data for electrochemically delithiated $FeSiO_4$ from $Li_2FeSiO_4$ indexed in space group *Pnma* (orthorhombic) with lattice constants $a$ = 10.3969(20) Å, $b$ = 6.5618(16) Å, and $c$ = 5.0334(8) Å.

| Atom | $g$ | $x$ | $y$ | $z$ | $100 \cdot U_{iso}$ |
|---|---|---|---|---|---|
| Fe | 0.5 | 0.6604(3) | 0.5158(7) | 0.2316(13) | 0.37(5) |
| Si | 1.0 | 0.4180(5) | 0.25 | 0.2903(12) | 0.22(8) |
| O1 | 1.0 | 0.4071(11) | 0.25 | 0.6154(14) | 0.62(35) |
| O2 | 1.0 | 0.5640(8) | 0.25 | 0.2445(42) | 0.68(31) |
| O3 | 1.0 | 0.3489(8) | 0.0675(7) | 0.1799(14) | 0.94(26) |

$R_{wp}$=1.69%    $R_p$=1.08%    $\chi^2$=1.19

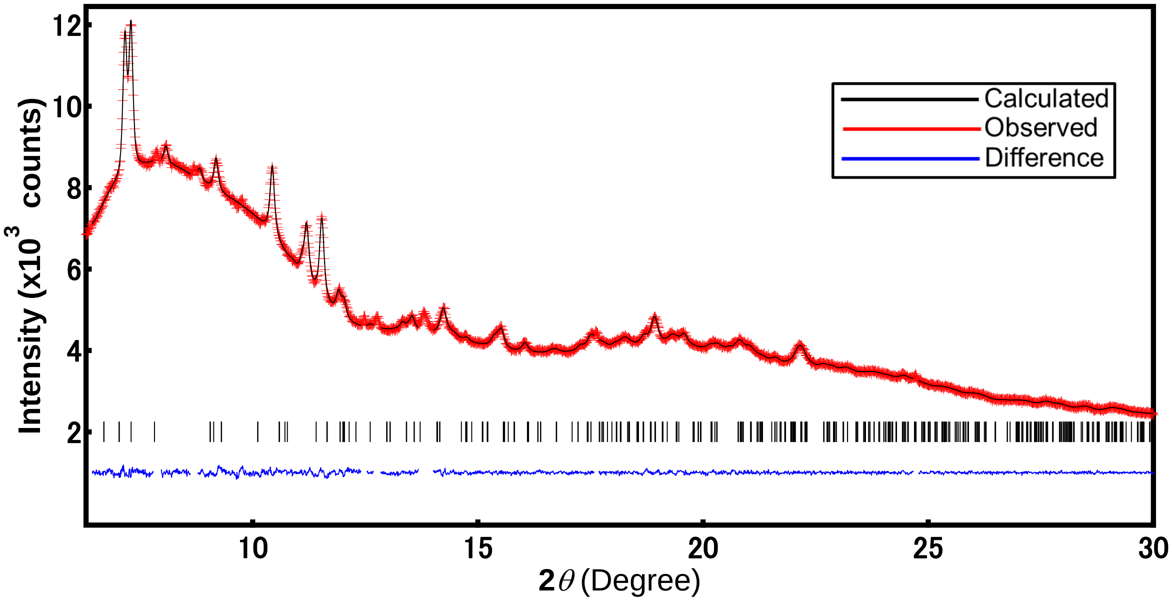


Fig. S8. Rietveld refinement pattern of synchrotron XRD data for electrochemically demagnesiated $FeSiO_4$ from $MgFeSiO_4$. Peaks that could not be properly indexed to the orthorhombic cell symmetry are excluded from the refinement.

Table S3. Atomic coordinates, occupancies, and atomic displacement parameters obtained by Rietveld refinement of synchrotron XRD data for electrochemically demagnesiated $FeSiO_4$ from $MgFeSiO_4$ phase indexed in space group *Pnma* (orthorhombic) with lattice constants: *a* = 10.3434 (19) Å, *b* = 6.5779 (13) Å, and *c* = 5.0185 (8) Å.

| Atom | *g* | *x* | *y* | *z* | $100 \cdot U_{iso}$ |
|---|---|---|---|---|---|
| Fe | 0.5 | 0.6538(3) | 0.4971(5) | 0.2237(10) | 0.53(7) |
| Si | 1.0 | 0.4208(4) | 0.25 | 0.2924(13) | 0.13(11) |
| O1 | 1.0 | 0.4171(9) | 0.25 | 0.6248(16) | 0.71(37) |
| O2 | 1.0 | 0.5473(7) | 0.25 | 0.2167(31) | 1.61(33) |
| O3 | 1.0 | 0.3575(7) | 0.0648(7) | 0.1869(16) | 0.39(23) |

$R_{wp}$=2.16%  $R_p$=1.55%  $\chi^2$=1.18

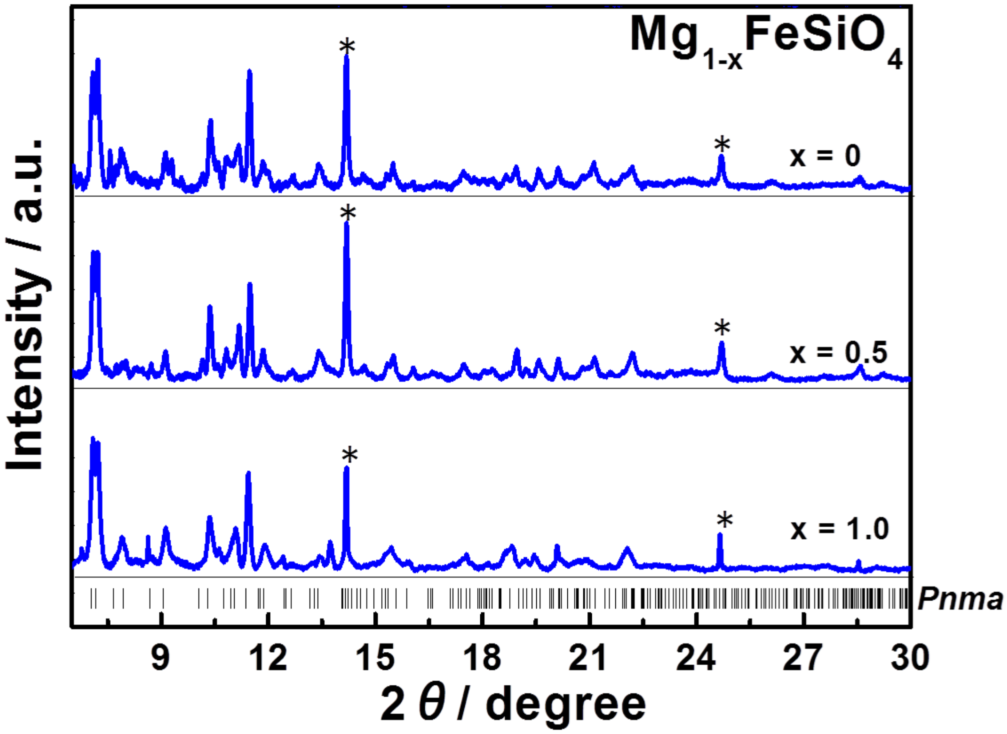


Fig. S9. Synchrotron XRD patterns of $Mg_{1-x}FeSiO_4$ during initial $Mg^{2+}$ insertion into the electrochemically delithiated $FeSiO_4$. The wavelength used was 0.50005 Å. Fe impurity peaks are indicated with asterisks.

## Theoretical potential by density functional theory calculations

Theoretical redox potential for the Mg insertion process of $Mg_xFeSiO_4$ was estimated by density functional theory (DFT) calculations as follows. XRD analyses of $Mg_xFeSiO_4$ indicate that Fe ions occupy half of the 8*d* sites in an orthorhombic unit cell ($Z = 4$) of a centrosymmetric *Pnma* space group. As the precise atomic arrangements of Fe ions are not clear, the arrangements in $MgFeSiO_4$ and $FeSiO_4$ were first addressed. The *Pnma* symmetry has seven maximal non-isomorphic subgroups that divide the 8*d* sites into two sets of 4-fold sites, and thus, the corresponding Fe arrangements were examined. $FeSiO_4$ exhibits a relatively large energy difference (3.25 eV per unit cell between the highest and lowest energies), whereas $MgFeSiO_4$ shows a small energy difference (0.13 eV per unit cell). The little influence of the Fe arrangement on energy can be ascribed to the same oxidation states and similar ionic radii of Fe and Mg ions. The Fe arrangements, shown in Fig. S10, show almost the same energies and they are lowest in energy for both $MgFeSiO_4$ and $FeSiO_4$.

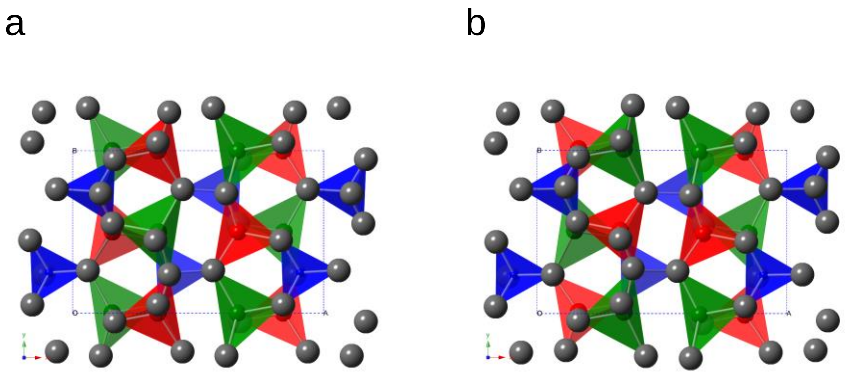


Fig. S10. Schematic atomic arrangements having lowest energies with (a) $Pna2_1$ and (b) $P2_1/n$ space group symmetries, respectively. Red, green and blue tetrahedra denote $FeO_4$, $MgO_4$ and $SiO_4$, respectively. Grey balls denote oxygen.

On the basis of these results, the arrangement of Fe ions in $Mg_{0.5}FeSiO_4$ was fixed as the abovementioned two stable arrangements. Thereafter, three choices were available for Mg arrangement within the unit cell (two ions at four sites) for each Fe arrangement. Thus, all possible arrangements of the unit cell of $Mg_{0.5}FeSiO_4$ gave six configurations, and the configuration with the lowest total energy was employed.

The theoretical potential, $E$, was estimated as $E(x_1 \leq x \leq x_2) = -(E^{DFT}[Mg_{x2}FeSiO_4] - E^{DFT}[Mg_{x1}FeSiO_4] - (x_2 - x_1) E^{DFT}[Mg])/2(x_2 - x_1) e$, where $E^{DFT}$ is the energy per formula unit estimated by the DFT calculations. The theoretical potential between the $MgFeSiO_4$ and $Mg_{0.5}FeSiO_4$ regimes, $E$ $(0.5 \leq x \leq 1)$, was 2.65 V *vs.* $Mg^{2+}/Mg$, whereas that between the $Mg_{0.5}FeSiO_4$ and $FeSiO_4$ regimes, $E$ $(0 \leq x \leq 0.5)$, was 3.84 V *vs.* $Mg^{2+}/Mg$. The theoretical potentials coincide with the experimental results, particularly for the charging voltage. The experimental voltage at the initial discharging seems rather low than the theoretical potential, and one would need precise electrochemical measurements of the reversible potential as well as sophisticated exchange-correlation interaction for oxygen states in the DFT calculations for a more detailed comparison.

The DFT calculations were performed using the plane-wave basis projected-augmented-wave (PAW) method implemented in the Vienna Ab initio Simulation Package (VASP) code. The plane-wave basis set was determined with a cut-off energy of 500 eV. Integral in the reciprocal space was evaluated by the Gaussian smearing technique with a smearing parameter of 0.1 eV and a $2 \times 4 \times 4$ mesh. Spin polarization was considered with the ferromagnetic spin

arrangements. The generalized gradient approximation with the Hubbard model correction (GGA+U) was employed for the exchange-correlation interaction with a U parameter of 5 eV for Fe-3*d* states. The atomic positions and lattice constants were optimized until the residual forces and stresses respectively became smaller than 0.02 eV $Å^{-1}$ and 2 GPa.

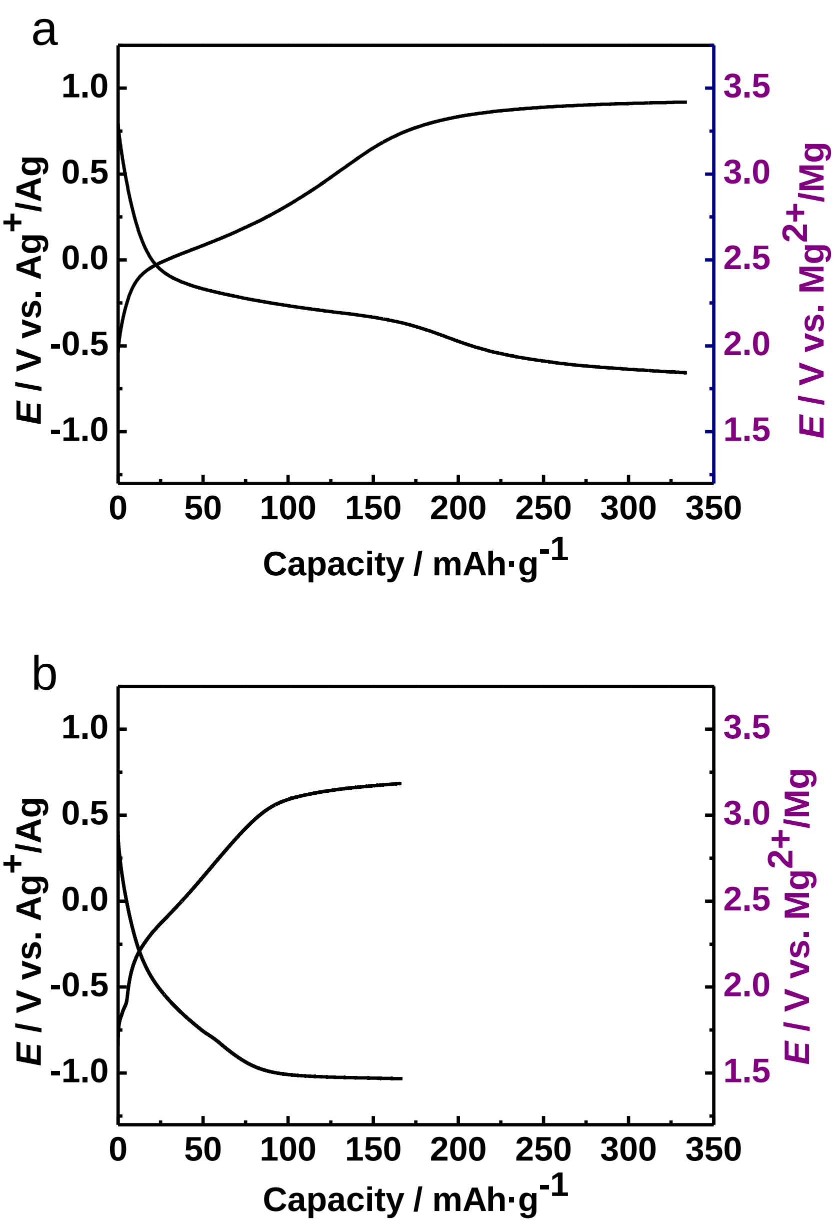


Fig. S11. Comparison of the voltage charge–discharge profiles of ion-exchanged $MgFeSiO_4$ using $Mg(TFSI)_2$ electrolyte in different solvents. (a) Three-electrode cells using Mg metal counter electrode and silver reference electrode were used. Electrolyte was 0.5 M magnesium (trifluoromethylsulfonyl)imide ($Mg(TFSI)_2$) in acetonitrile (solvent). Measurement temperature was 55°C. Current density was 6.62 $mA \cdot g^{-1}$ ($MgFeSiO_4$). (b) Three-electrode cells using Mg metal counter electrode and lithium reference electrode were used. Electrolyte was 0.5 M

magnesium (trifluoromethylsulfonyl)imide ($Mg(TFSI)_2$) in triglyme (solvent). Measurement temperature was 100°C. Current density was 6.62 $mA{\cdot}g^{-1}$ ($MgFeSiO_4$). Capacity range was limited to 0.5 $Mg^{2+}$ per Fe. Compared with Fig. S11(a), which corresponds to the use of $Mg(TFSI)_2$ in acetonitrile solvent, higher polarization is observed using triglyme as solvent.

References:


1. A. R. Armstrong, N. Kuganathan, M. S. Islam, P. G. Bruce, *J. Am. Chem. Soc.* **133**, 13031-13035 (2011).
